# Neuroprotective Effect of Paroxetine on Memory Deficit Induced by Cerebral Ischemia after Transient Bilateral Occlusion of Common Carotid Arteries in Rat

**Published:** 2018

**Authors:** Yazdan Naderi, Siavash Parvardeh, Taraneh Moini Zanjani, Masoumeh Sabetkasaei

**Affiliations:** *Department of Pharmacology, School of Medicine, Shahid Beheshti University of Medical Sciences, Tehran, Iran.*

**Keywords:** Paroxetine, Memory, Morris Water Maze, Cerebral ischemia, Neuroprtection

## Abstract

Memory deficit is the most visible symptom of cerebral ischemia. The hippocampus is sensitive against cerebral ischemia. Oxidative stress and inflammation are involved in the pathological process after cerebral ischemic injury. Paroxetine has anti-oxidative and anti-inflammatory effects. In this study, the effect of paroxetine on memory deficit after cerebral ischemia was investigated. Cerebral ischemia/reperfusion (I/R) injury model was established using the bilateral occlusion of common carotid artery method. Paroxetine (10 mg/kg) was intraperitoneally injected into rats, 24 h before surgery or once a day for 7 days after surgery. Learning and memory were evaluated using the Morris water maze task, then the brain tissue was fixed and hippocampal CA1 pyramidal cells damage was analyzed using the Nissl staining method. In the ischemia group the escape latency time (ELT) and the swimming path length (SPL) were significantly increased and the time spent in target quadrant (TSTQ) was significantly decreased compared with the control group. The ELT and the SPL were significantly shortened and the TSTQ was significantly increased compared with the ischemia group after Pre- or post-ischemic administration of paroxetine. The percentage of viable pyramidal cells in the ischemia group was significantly decreased compared with the control group. The percentage of viable cells was significantly increased following pre-or post-ischemic administration of paroxetine compared with the ischemia group. Memory deficit due to I/R was improved and the percentage of viable cells in CA1 region was increased after administration of paroxetine. Therefore, paroxetine may have a neuroprotective effect against cerebral ischemia**.**

## Introduction

Stroke is defined as the disturbance of the cerebral blood flow and is the most common cause of death and disability (1-4). Stroke could result in the cognition impairment and the prevalence of post-stroke cognitive impairment ranges from 20% to 80% (2, 5, 6). Transient bilateral occlusion of common carotid arteries (BOCCA) is considered to be a reliable rodent model of cerebral ischemia (7, 8, 9). Clinical and experimental evidence suggest a causal relationship between hippocampal damage and the development of cognitive deficits and dementia (10-12). BOCCA causes selective vulnerability and delayed neuronal cell death in several regions of the brain (13). Among them, the CA1 subfield of the hippocampus has attracted interest for the investigation of delayed neuronal death because it was reported to be the most vulnerable region to ischemia insults (14-16). Oxidative stress and inflammation after ischemia/reperfusion (I/R) impair spatial learning and memory due to death of CA1 cells in the hippocampus (17-20). Paroxetine, a common selective serotonin reuptake inhibitor (SSRI), is widely presented as an antidepressant because it has fewer side effects and lower toxicity than earlier generation of SSRIs (21). Antidepressant such as fluoxetine and imipramine have been shown to possess potent anti-inflammatory effects in the rat cerebral ischemia model of middle cerebral artery occlusion (22-25). SSRIs have also been found to stimulate nerve growth in the hippocampus and chronic fluoxetine treatment that can enhance neurogenesis and benefits spatial cognitive function recovery following ischemia insults (24).These studies demonstrated that fluoxetine and imipramine attenuated expression of tumor necrosis factor alpha (TNF-α) and interleukin 1 beta (IL-1β) (24). Similarly paroxetine was found to decrease the release of TNF-α in rat splenocytes (21, 25, 26). Paroxetine possesses anti-inflammatory properties and inhibits glial activation-mediated oxidative stress. Therefore, paroxetine has therapeutic value in the treatment of neurodegenerative disease (21, 26, 27). The purpose of the current study was to evaluate the effects of paroxetine in memory deficit and the number of viable cells after cerebral ischemia due to BOCCA in rats.

## Experimental

Animals**.** All of the experiments were done in accordance with approved animal protocol and guidelines established by ethics committee of Shahid Beheshti University of Medical Sciences. Sixty male Wistar rats (250-300g) were obtained from animal house of pharmacology group of Shahid Beheshti University of Medical Sciences. Male Wistar rats were housed in rodent cages in the animal room, maintained at 22-24 ºC on a 12 h light/dark cycle. The animals were allowed ad libitum access to food and water throughout the study.

Drug preparation. Paroxetine hydrochloride (Sigma, U.S.A) was dissolved in normal saline. Ketamine hydrochloride and xylazine hydrochloride (both from Sigma, U.S.A) were used for anesthesia. All drugs were injected by the (i.p). route.

Experimental groups. Two methods were performed for drug administration: 1- Pre-ischemic administration: paroxetine (10 mg/kg) or normal saline (vehicle) was injected 24 h before surgery by the )i.p(. route 2- Post-ischemic administration: paroxetine (10 mg/kg) or normal saline was injected once a day for 7 days (day 0 to 6) after surgery by the )i.p(. route. Sixty adult male Wistar rats were divided randomly into six groups (10 rats in each group) (3 groups for pre-ischemic and 3 groups for post-ischemic administration of paroxetine or vehicle). Ischemia group: BOCCA method was performed for 20 min. Normal saline was injected i.p., 24 h before I/R (pre-ischemic) or once a day during 7 days after I/R (post-ischemic). Control group: All surgical steps were performed except occlusion of common carotid arteries. Normal saline solution was injected )i.p(, 24 h before surgery (pre-ischemic) or once a day during 7 days after surgery (post-ischemic).Treatment or prevention group: BOCCA was performed for 20 min. Paroxetine (10 mg/kg) was injected )i.p(, 24 h before I/R (treatment) or once a day during 7 days after I/R (prevention) (21).

*Transient bilateral occlusion of common carotid artery*

The rats were anesthetized with administration of ketamine (100 mg/kg, i.p) and xylazine (10 mg/kg, i.p). Animals were placed in the supine position and both common carotid arteries were exposed by means of a ventral cervical incision. The arteries were separated from vagal nerves and occluded by vascular clamps for 20 min. In the control group animals underwent the same surgical procedure but without the occlusion. Reperfusion was introduced by releasing the clamps placed around the carotid arteries. After reperfusion, incision was sutured. Temperature was maintained around 37 ºC throughout the surgical procedure (28).

*Morris water maze*

Spatial learning and memory were measured using the Morris water maze task, 7 days after I/R. The circular pool was 150 cm in diameter and filled with water to a depth of 50 cm. The animals were released from each of 4 releasing points (S, N, E, W). The pool surface was divided into four quadrants of equal area (SE, SW, NE, NW). The escape platform was placed in the center of SE. The platform was set 1.5 cm below the water level. The rats could not see the platform because water in pool was turbid with milk. Behavior of the rat in the pool was recorded by a video camera, positioned on the ceiling in the center of the testing room. During a hidden platform trial the experimenter released the rat into the pool facing the pool wall. Rats were allowed to find the hidden platform during 60 sec and remain on the platform for 20 sec after finding it. If the platform was not found during 60 sec, the rat was guided to the platform and allowed to remain there for 20 sec. Animals received 4 consecutive days of training with hidden platform and each day included 4 trials with a 60 sec interval between trials. The order of release point was varied on each training trials and changed each day. Probe trial was performed for evaluation of spatial learning on fifth day, the platform was removed and animals were allowed to swim for 60 sec. Escape latency time (ELT), swimming path length (SPL) and swimming speed (SS), during trial days (day 1 to 4) and time spent in target quadrant (TSTQ) and SS in probe trial day (fifth day) were measured for each rat (29).

*Histopathology*

One day after Morris water maze task, animals were anasthetized with ketamine (100 mg/kg) and xylazine (10 mg/kg i.p) and pre-fixation was achieved by trans-cardiac perfusion with 150 mL normal saline followed by 200 mL of 4% paraformaldehyde (PFA) in 0.1 M phosphate buffer saline. The brains were removed, post-fixed in 4% PFA at 4 ºC. The samples were embedded in paraffin and 6 μm thickness sections were stained with Nissl method in witch the sections (3 sec of the hippocampus of each rat between the levels of 2.3 and 5 mm posterior to bregma fortune) were stained with 0.5% cresyl violet, dehydrated through graded alcohols (70, 80, 90 and 100 ˣ 2), placed in xylene and coverslipped. By a camera connected to the microscope (Olympus Ax70) images were taken at ×400 magnification then the number of intact an ischemic hippocampal CA1 pyramidal cells were counted using the semiquantitative visual method (30). 

*Statistical analysis*

Data were analyzed using Graphpad prism 6 and were presented as mean ± SEM. The data of different groups were compared using analysis of variance (ANOVA) with post hoc Tukey test for probe test (day 5) and Nissl staining. Two way (ANOVA) was used and the effect of two independent variables (day and group) on the dependent variable (escape latency time, swimming path length and swimming speed) was analyzed. Results were considered significantly different when *p* ˂ 0.05.

## Results

*The effects of pre-ischemic and post-ischemic administration of paroxetine on the escape latency time during trial days*

**Figure 1:**
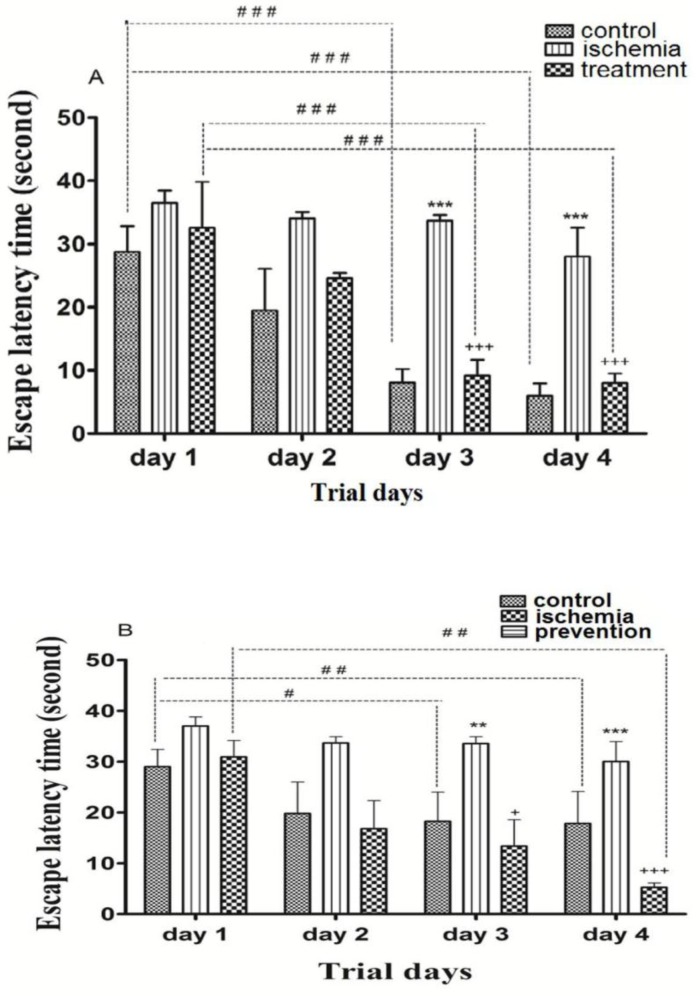
The effects of post-ischemic (A) or pre-ischemic (B) administration of paroxetine (10 mg/kg, i.p) on the escape latency time (in second) during trial days in rats. The escape latency time was significantly increased on day 3 and day 4 in the ischemia group compared with the control group (A, B). The escape latency time was significantly decreased on day 3 and day 4 in the treatment (A) and the prevention (B) groups compared with the ischemia group. Data are expressed as mean ± SEM (n = 10). (Compared with control group, **p < 0.01, ***p < 0.001; compared with ischemia group, + *p* < 0.05, +++ *p* < 0.001; compared with day 1, # *p* < 0.05, ## *p* < 0.01, ### *p* <0.001

Post-ischemic administration of paroxetine**.** The ELT in the ischemia group was significantly higher than that of the control group (*p *˂0.001). The ELT in the treatment group was significantly reduced by paroxetine administration (10 mg/kg) compared to the ischemia group (*p *˂0.001). The ELT was significantly reduced in the control group and treatment group on day3 and day 4 compared to day 1 (*p *˂0.001). (Figure 1A)

Pre-ischemic administration of paroxetine**.** The ELT in the ischemia group was significantly higher than that of the control group on day 3 *(p ˂0*.01) and day 4 (*p *˂0.001). The ELT in the prevention group was significantly reduced by paroxetine administration (10 mg/kg) compared to the ischemia group on day 3 (*p *˂0.05) and day 4 (*p *˂ 0.001). The ELT was significantly reduced in the control group on day 3 (*p *˂0.05) and day 4 (*p *˂0.01) compared to day 1. The ELT was significantly reduced in the prevention group on day 4 compared to day 1 (*p *˂0.01). (Figure 1B)

*The effects of pre-ischemic and post-ischemic administration of paroxetine on the swimming path length to reach the hidden escape platform*

**Figure 2:**
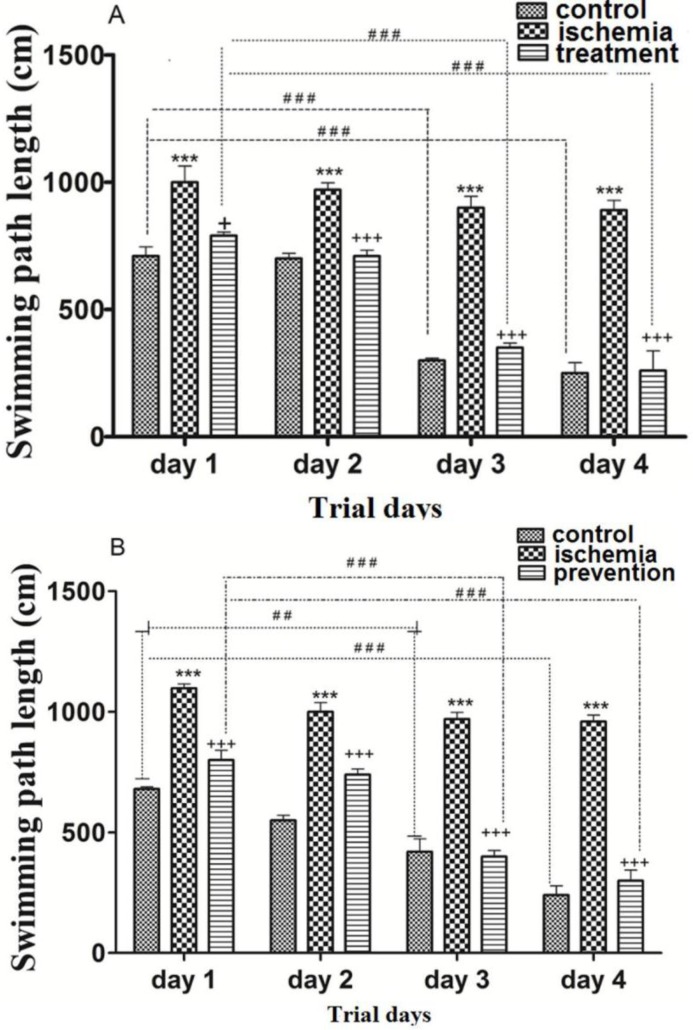
The effects of post-ischemic (A) or pre-ischemic (B) administration of paroxetine (10 mg/kg, i.p) on the swimming path length during trial days in rats. The swimming path length was significantly increased during trial days in the ischemia group compared with the control group (A, B). The swimming path length was significantly decreased during trial days in the treatment (A) and the prevention (B) groups compared with the ischemia group. Data are expressed as mean ± SEM (n = 10). (Compared to control group, *** *p* < 0.001; compared to ischemia group, + *p* < 0.01, +++ *p* < 0.001; compared to day 1, ## *p* < 0.01, ###*p* < 0.001

Post-ischemic administration of paroxetine**.** The SPL in the ischemia group was significantly higher than that of the control group during trial days (first to forth days) (*p *˂0.001). The SPL in the treatment group was significantly reduced by paroxetine administration (10 mg/kg) compared to the ischemia group on day 1 (*p *˂0.05) and days 2, 3 and 4 (*p *< 0.001). The SPL was significantly shortened in the control and the treatment groups on day 3 and day 4 compared to day 1 (*p *<0.001). (Figure 2A)

Pre-ischemic administration of paroxetine**.** The SPL in the ischemia group was significantly higher than that of the control group during trial days (first to forth days) (*p *<0.001). The SPL was significantly reduced by paroxetine administration (10 mg/kg) in comparison with the ischemia group (*p *<0.001). The SPL was significantly reduced in the control group on day 3 (*p *<0.01) and day 4 (*p *<0.001) compared to day 1. The SPL was significantly reduced in the prevention group on day 3 and day 4 compared to day 1 (*p *<0.001). (Figure 2B)

*The effects of pre-ischemic and post-ischemic administration of paroxetine on the swimming speed during trial days (first to forth days) and probe trial day (fifth day)*

**Figure 3:**
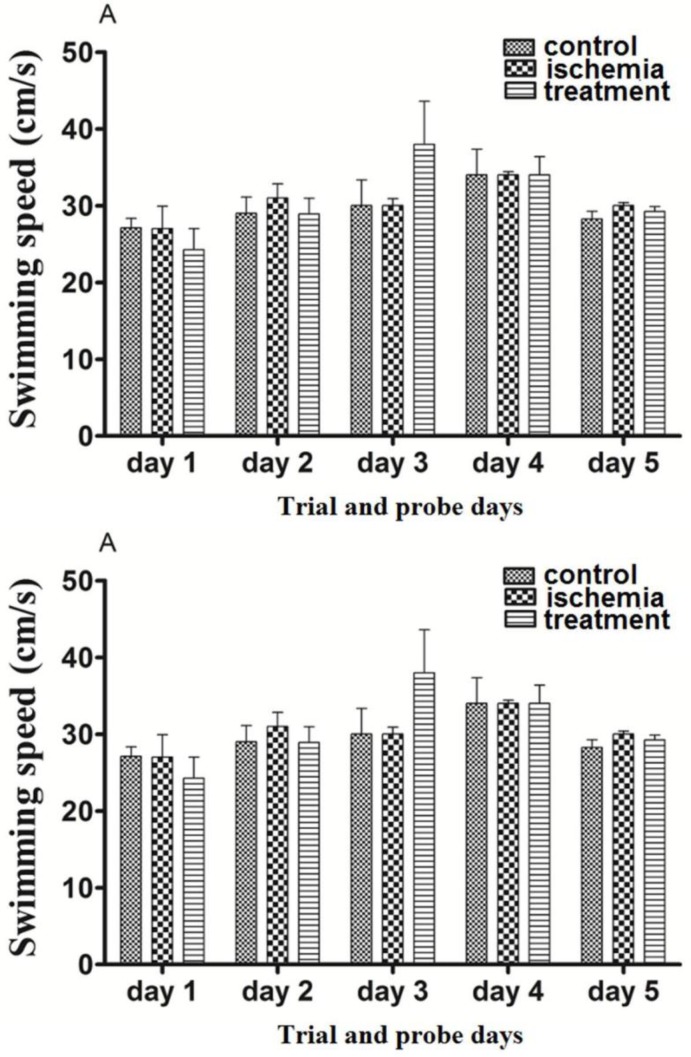
The effects of post-ischemic (A) or pre-ischemic (B) administration of paroxetine (10 mg/kg, i.p) on the swimming speed during trial days and probe trial day in rats. Animals in different groups did not show significant change in their swimming speed (*p* > 0.05). Data are expressed as mean ± SEM (n = 10

Post-ischemic administration of paroxetine**.** The SS in the ischemia group was not significantly changed in comparison with the control group at first to fifth days (trial and probe test days) (*p *>0.05). The SS in the treatment group was not changed significantly by paroxetine administration (10 mg/kg) in comparison with the ischemia group on the first to fifth days (*p *>0.05)The SS was not changed during the first 4 days (trial days) and the fifth day (probe test) compared to day 1 (*p *>0.05). (Figure 3A)

Pre-ischemic administration of paroxetine**.** The SS in the ischemia group was not significantly changed in comparison with the sham group on the first to fifth days (trial and probe test days) (*p *>0.05). The SS in the prevention group was not changed significantly by paroxetine administration (10 mg/kg) in comparison with the ischemia group on the first to fifth days (*p *>0.05) The SS was not changed during the first 4 days (trial days) and the fifth day (probe test) compared to day 1 (*p *>0.05). (Figure 3B)

*The effects of pre-ischemic and post-ischemic administration of paroxetine on the time spent in target quadrant in probe test day (fifth day)*

**Figure 4:**
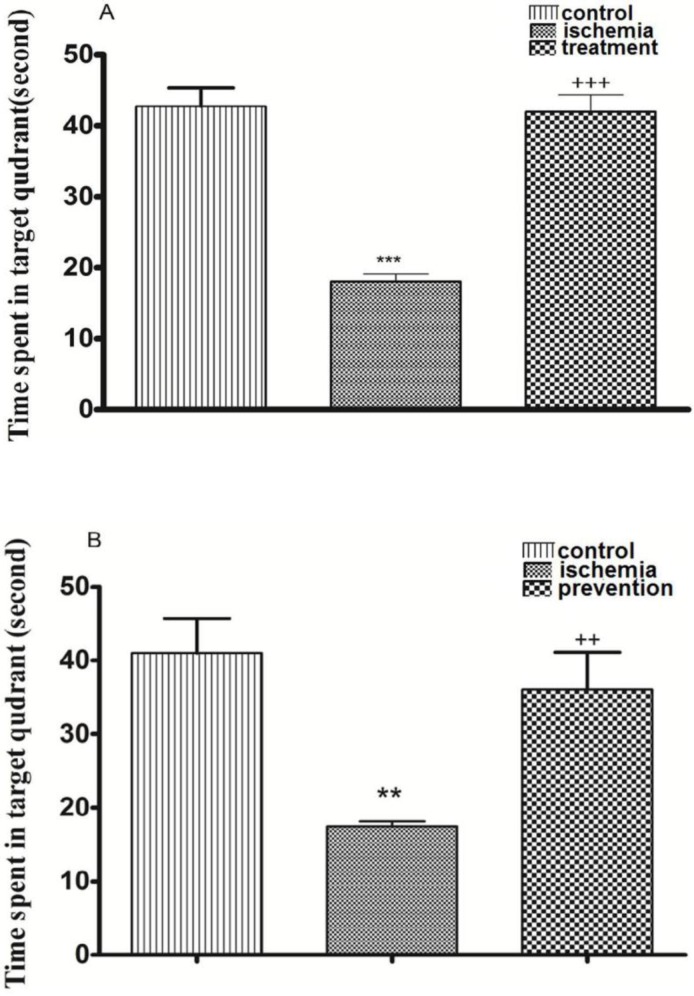
The effects of post-ischemic (A) or pre-ischemic (B) administration of paroxetine (10 mg/kg, i.p) on the time spent in target quadrant in probe trial day (fifth day). The time spent in target quadrant was significantly decreased in the ischemia group compared with the control group. The time spent in target quadrant was significantly increased in the treatment (A) and the prevention (B) groups compared with the ischemia group. Data are expressed as mean ± SEM (n = 10). (Compared with control group, ** *p* < 0.01, *** *p* < 0.001; compared with ischemia group, ++ *p* < 0.01, +++ *p* < 0.001)

Post-ischemic administration of paroxetine. The TSTQ in the ischemia group was significantly decreased in comparison with the control group on the fifth day (probe test) (*p *<0.001). The TSTQ in the treatment group was significantly increased by paroxetine administration (10 mg/kg) in comparison with the ischemia group (*p *<0.001). (Figure 4A)

Pre-ischemic administration of paroxetine**.** The TSTQ in the ischemia group was significantly reduced in comparison with the control group (*p *<0.01). The TSTQ in the prevention group was significantly increased by paroxetine administration (10 mg/kg) in comparison with the ischemia group (*p *<0.01). (Figure 4B)

*The effects of pre-ischemic and post-ischemic administration of paroxetine on the percentage of viable CA1 region cells of the hippocampus*

**Figure 5:**
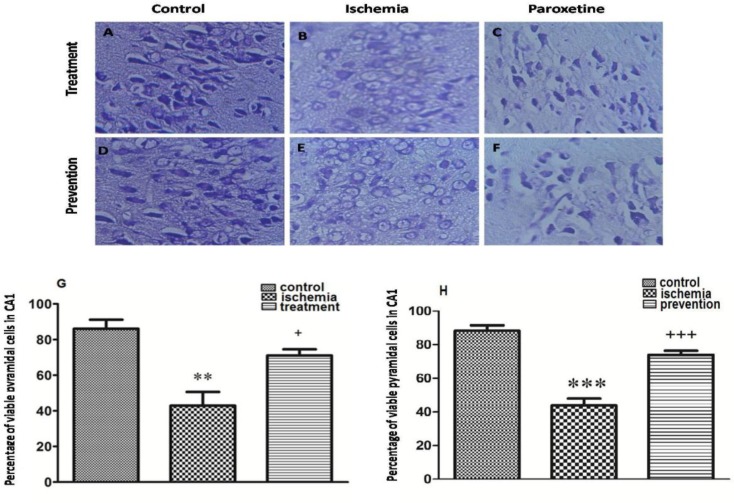
Effect of pretreatment or treatment with paroxetine on hippocampal pyramidal cells at 7 days after reperfusion. The morphological changes of neuronal cells in CA1 region of the hippocampus were observed by a microscope (A-F, scale bar = 100μm; magnification ×400). The neuronal damage was increased in the ischemia groups (B, E) compared with the control groups (A, D). Also the neuronal damage was decreased in the treatment(C) and the prevention (F) groups compared with the ischemia groups (B, E). The percentage of viable pyramidal cells in CA1 region was significantly decreased in the ischemia group compared with the control group (G, H). Also the percentage of viable pyramidal cells in CA1 region was significantly increased in the treatment (G) and the prevention (H) groups compared with the ischemia group. Data are expressed as mean ± SEM (n = 10). (Compared with control group, ** *p* < 0.01, *** *p* < 0.001; compared with ischemia group, + *p* < 0.05, +++ *p* < 0.001

Post-ischemic administration of paroxetine**.** The percentage of viable CA1 region cells of the hippocampus was significantly decreased in the ischemia group (Figure 5B) compared to that of the control group (*p *<0.01) (Figure 5A). The percentage of viable cells in the treatment group was significantly increased compared to that of the ischemia group after post-ischemic administration of paroxetine (10 mg/kg) (*p *<0.05) (Figure 5G, 5C).

Pre-ischemic administration of paroxetine**. **The percentage of viable CA1 region cells of the hippocampus was significantly decreased in the ischemia group (Figure 5E) compared to that of the control group (*p *<0.001) (Figure 5D). The percentage of viable cells in the prevention group was significantly increased compared to that of the ischemia group after pre-ischemic administration of paroxetine (10 mg/kg) (*p *<0.001) (Figure 5H, 5F).

## Discussion

In the present study, the results showed that paroxetine has neuroprotective effect and prevents cognitive impairments due to ischemia/reperfusion. This is the first report that investigates the effect of paroxetine on ischemia/reperfusion injury due to transient occlusion of common carotid arteries in rats.

Previous studies have revealed that the hippocampus and cortex of rats are the most vulnerable brain structure affected by ischemia (16, 19). Inflammation, oxidative stress, and apoptosis are involved in the pathological process after I/R injury and cause damage of the hippocampal neurons (18-20). I/R increases the activity of the microglia cells and initiates an inflammatory response involving the expression of inflammatory cytokines such as TNF-α and IL-1β in ischemic regions of the brain (31-33). The activation and overexpression of cyclooxygenase-2 (COX-2) play an important role in relation to the pathology of I/R injury (19, 31, 32, 33). Also, the increased oxidative stress after cerebral ischemia leads to apoptosis and cell death in CA1 pyramidal neurons of the hippocampus (12, 20, 34). 

Many studies have indicated that serotonergic system plays an important role in cognitive function by interacting with cholinergic and glutamatergic systems (35-37). CA1 pyramidal cells of the hippocampus express various types of serotonin receptors (5-HTR) such as 5-HT_1A_, 5-HT_4_ and 5-HT_7_ that have important role in memory and learning (37). Previous studies in rodents have demonstrated that stimulation of 5-HT_1A_ receptors produces memory impairment (35). In contrast, blockade of 5-HT_1A_ receptors facilitates memory by enhancing hippocampal cholinergic and glutamatergic neurotransmission (35, 36). However, long term use of SSRIs can lead to memory impairment because of their effects on serotonin receptors. There are several evidences suggesting that SSRIs such as fluoxetine have neuroprotective effect after cerebral ischemia and improve spatial learning and memory following I/R because of their anti-inflammatory and antioxidant effects (23-25). 

Paroxetine, a common serotonin reuptake inhibitor, is widely prescribed as an antidepressant. Previous studies have shown that it has anti-inflammatory and antioxidative effects and decreases release of inflammatory cytokines from microglia cells (25-27). Also, paroxetine has free radical scavenging properties and decreases oxidative stress due to ischemia (25, 27). Therefore, the purpose of the current study was to establish a transient cerebral ischemia/reperfusion rat model and evaluate the neuroprotective effect of paroxetine on the transient bilateral occlusion of common carotid arteries model.

Transient BOCCA is a model of I/R, was used to impair spatial learning and memory in rats ([9, 13, 28). Ischemic-induced cell death in CA1 pyramidal neurons of the hippocampus causes spatial learning and memory deficits that are the most visible symptoms of the BOCCA (6-8). Seven days after reperfusion spatial learning and memory were evaluated by Morris water maze method. Consistent with our findings, many studies demonstrated that antioxidant and anti-inflammatory substances have neuroprotective effect on memory deficit induced by I/R (18-20). The results of this study demonstrated that the ELT and SPL in the ischemia group were significantly increased by I/R compared with the control group and the TSTQ in the ischemia group was significantly decreased by I/R compared with the control group in the probe test day (fifth day). These results showed that spatial learning and memory were impaired by I/R in the ischemia group in rats. In this study the ELT and SPL in the treatment and the prevention groups were significantly decreased and the TSTQ was significantly increased in comparison with the ischemia group. These results showed that spatial learning and memory deficits after I/R were improved following pretreatment or treatment with paroxetine (pre-ischemic or post-ischemic administration). The SS of rats during the first 4 days (trial days) and the fifth day (probe test) did not change after treatment or pre-treatment with paroxetine in the treatment and the prevention groups and I/R in the ischemia group. Therefore, movement function was not impaired by I/R and paroxetine administration in rats during trial days and probe day (first to fifth days) because animals in different groups did not show significant change in their swimming speed. 

The hippocampus is highly sensitive to ischemia injury, particularly the pyramidal neurons in the hippocampal CA1 region. Cell death can be found 3-4 days after I/R and reach to a peak 7 days after I/R (6-8). Inflammation, oxidative stress, and apoptosis play an important role in neuronal damage following I/R (21, 22, 26). Previous study revealed that antioxidant and anti-inflammatory substances can prevent neuronal death due to I/R in CA1 region of the hippocampus (19, 20). In this study, the percentage of viable cells was measured by Nissl staining method 7 days after reperfusion and the results showed that cell death in CA1 region of the hippocampus due to I/R injury was decreased following pre-ischemic or post-ischemic administration of paroxetine. Therefore, the results of this study demonstrate that paroxetine has neuroprotective effect in rats with cerebral I/R-induced injury. 

## Conclusion

This study demonstrated that paroxetine had neuroprotective effect on cerebral ischemia/reperfusion injury. It prevented cognitive impairment in ischemia induced rats and reduced death of hippocampal pyramidal cells.
